# The Effect of Hyperoxia on Nitric Oxide Metabolism in the Skeletal Muscle of Male Type 2 Diabetic Rats

**DOI:** 10.1002/edm2.70090

**Published:** 2025-08-04

**Authors:** Mahdis Mousavi, Sajad Jeddi, Reza Norouzirad, Asghar Ghasemi

**Affiliations:** ^1^ Endocrine Physiology Research Center, Research Institute for Endocrine Molecular Biology, Research Institute for Endocrine Sciences Shahid Beheshti University of Medical Sciences Tehran Iran; ^2^ Department of Biochemistry, School of Medicine Dezful University of Medical Sciences Dezful Iran

**Keywords:** arginase, hyperoxia, nitric oxide, type 2 diabetes

## Abstract

**Introduction:**

Hypoxia is involved in the pathophysiology of type 2 diabetes (T2D), and oxygen therapy (hyperoxia) has been proposed for managing T2D. As a side effect, hyperoxia increases nitric oxide (NO) metabolism and decreases NO bioavailability. This study aims to investigate the effect of hyperoxia on NO synthases (NOSs), which produce NO from *L*‐arginine and arginase, which degrades *L*‐arginine in the soleus muscle (SM) of rats with T2D.

**Methods:**

A combined high‐fat diet and a low dose of streptozotocin (30 mg/kg) were used to induce T2D in rats. Rats with T2D were divided into four groups (*n* = 6/group): Control rats exposed to normoxia (Control), control rats exposed to hyperoxia (C + HOX), diabetic rats exposed to normoxia (T2D) and diabetic rats exposed to hyperoxia (T2D + HOX). The hyperoxia and the control groups received 95% and 21% oxygen for 35 days, respectively. SM was isolated at day 35, and the protein levels of endothelial NOS (eNOS), inducible NOS (iNOS), arginase, as well as tissue concentrations of lactate and NO metabolites (nitrate+nitrite = NOx) were measured.

**Results:**

Compared to T2D, T2D + HOX rats had lower lactate concentration by 38% (*p* = 0.009) and NOx concentration by 23% (*p* = 0.011) in SM. In SM of rats with T2D, hyperoxia decreased eNOS protein by 46.2% (1.4 ± 0.13 vs. 2.6 ± 0.2 ng/mg protein, *p* = 0.002) and increased arginase protein by 2.3‐fold (1.04 ± 0.05 vs. 0.31 ± 0.07 ng/mg protein, *p* < 0.001) but did not affect iNOS protein. Hyperoxia did not affect lactate concentration, eNOS and iNOS in SM of control rats but decreased NOx concentration by 25% (*p* = 0.003).

**Conclusion:**

Hyperoxia decreased NO bioavailability in SM of rats with T2D; this effect was associated with decreased eNOS and increased arginase protein levels. These findings suggest that oxygen therapy in diabetic rats may decrease NO bioavailability as a potential side effect.

## Graphical abstract

Hyperoxia decreased nitric oxide (NO) bioavailability in soleus muscle of rats with type 2 diabetes; this effect was associated with decreased NO metabolites (NOx) and endothelial NO synthases (eNOS), and increased arginase protein levels.

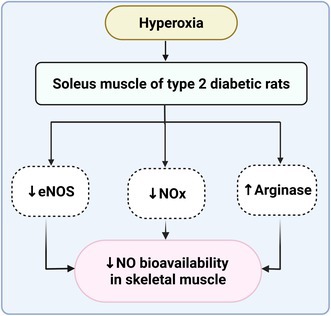

## 1 Introduction

Diabetes is among the top 10 causes of death, with 3.4 million deaths registered in 2024 among adult subjects with diabetes [1]. According to the 2025 reports of the International Diabetes Federation (IDF), the worldwide prevalence of diabetes was 11.1% (589 million), and it is estimated to rise to 13% (835 million) by 2050 [1]. Type 2 diabetes (T2D) constitutes over 90% of all diabetes cases worldwide [1]. Despite available oral anti‐diabetic medications for T2D, 60% of patients do not have optimal glycaemic control (glycosylated haemoglobin (HbA1c) < 7%) [2, 3]. In addition, by increasing the duration of T2D, achieving optimal glycaemia with oral anti‐diabetic drugs is more difficult due to drug failure, and insulin therapy is needed in most cases [3]. This issue warrants the need to develop new treatment modalities for T2D.

In addition to insulin resistance and beta‐cell dysfunction, which make the core pathophysiology of T2D, hypoxia plays a significant role in developing T2D [4]. Hypoxia increases anaerobic metabolism, decreases ATP production and ultimately leads to cell death [5]. Compared to non‐diabetic subjects, reduced blood oxygen saturation has been reported in patients with type 1 diabetes (T1D) [6, 7] and T2D [8]. In addition, compared to healthy subjects, partial pressure of oxygen in the skeletal muscle is lower in obese men (10–20 mmHg vs. 27–30 mmHg [9, 10]), which may be involved in the pathophysiology of T2D. Therefore, hyperoxia has been proposed for managing T2D [5], and its favourable metabolic effects have been reported in rats [11, 12] and obese patients [13] with T2D.

Nitric oxide (NO) is generated from *L*‐arginine by NO synthases (NOS) enzymes, of which three isoforms, including neuronal NOS (nNOS), inducible NOS (iNOS) and endothelial NOS (eNOS), have been identified [14]. T2D is associated with decreased NO availability because of lower *L*‐arginine transport, uncoupling of NOS enzymes, increased arginase activity and elevated levels of asymmetric dimethyl‐*L*‐arginine (ADMA) [14]. In addition, eNOS and nNOS expressions are decreased, but iNOS expression increased in the liver, adipose tissue and soleus muscle (SM) of rats with T2D [15]. Hyperoxia further decreases NO bioavailability in T2D [16, 17] by enhancing arginase activity [18, 19] and oxidative stress‐induced NO inactivation [14]. The effect of hyperoxia on NOS expression is inconsistent; decreased [14, 20, 21] and no change in eNOS expression as well as decreased [22, 23], increased [24] and no change [18, 19, 20, 21] in iNOS expression have been reported. Thus, reduced NO bioavailability is a side effect of oxygen therapy in T2D, which itself is a state of NO deficiency.

Previous studies reported time‐ and tissue‐dependent responses to hyperoxia; long‐term exposure (35 days) reduces eNOS levels [20], whereas short‐term exposure (3.5 days) has no effect [18]. In addition, the effects of hyperoxia on NO metabolism in lung [19, 21, 24, 25], liver [18], heart [23] and eye [22] have been addressed in healthy animals subjected to short‐term hyperoxia for 1 [23], 2 [25], 2.5 [19, 24], 3.5 [18], 5 [21] and 7 [22] days. Hyperoxia does not affect eNOS in the liver [18] but decreases it in the lung [21]. The effects of hyperoxia on NO metabolism in the skeletal muscle, which is the largest body tissue contributing to 80%–90% of insulin‐stimulated glucose uptake [4], have not been reported previously. Among skeletal muscles, SM is preferred for studying metabolic changes in diabetes as it contains a high proportion (~95%) of type I (slow‐twitch) fibres [26], which are characterised by high mitochondrial density and oxidative capacity [27]. This study aims to determine the effect of hyperoxia on NO‐producing enzymes (eNOS and iNOS) and *L*‐arginine degrading enzyme (arginase) in the SM of obese male rats with T2D.

## 2 Material and Methods

### 2.1 Animals and Ethics

According to the principle of the 3Rs (Replacement, Reduction and Refinement [28]), to reduce the number of rats used in this study, serum and samples were sourced from our previous project in which the beneficial effects of hyperoxia on carbohydrate metabolism were reported [11]. All experimental procedures were approved by the ethics committee of the Research Institute for Endocrine Sciences affiliated with Shahid Beheshti University of Medical Sciences (IR.SBMU.ENDOCRINE.REC.1403.132) and were reported according to ARRIVE guidelines [29].

### 2.2 Study Design

**FIGURE 1:**
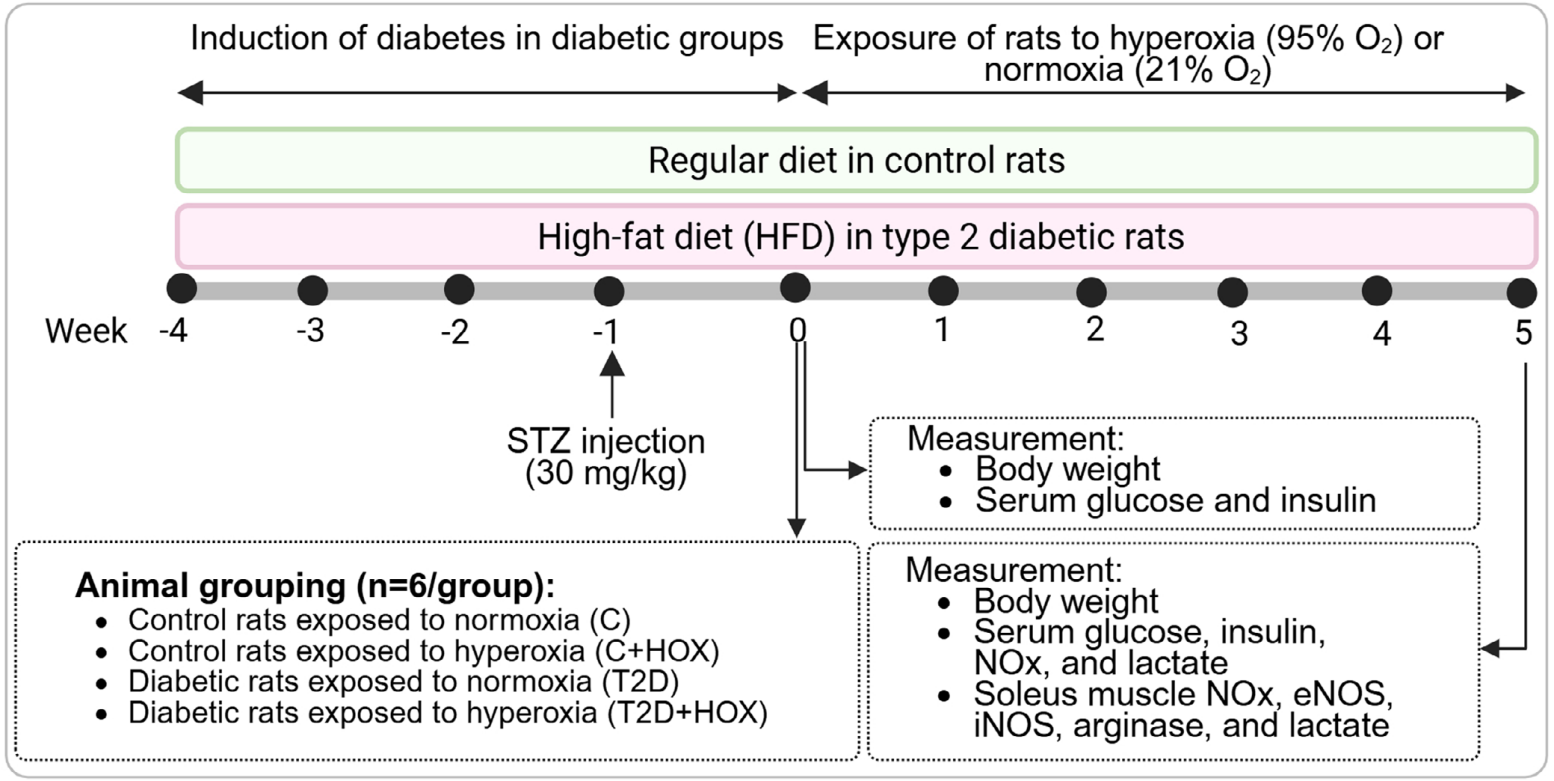
Experimental design of the study. T2D, type 2 diabetes; STZ, streptozotocin; NOS, nitric oxide synthase; eNOS, endothelial NOS; iNOS, inducible NOS; NOx, nitric oxide metabolites. Created with BioRender.com.

The design of this interventional experimental study is illustrated in Figure 1. This experimental study used 24 male Wistar rats with a body weight range of 190–210 g. After induction of T2D (week −4 to week 0), rats were randomly assigned to four groups (*n* = 6/group) at week 0 using the random function of the Excel program as described in detail previously [30]: Control rats exposed to normoxia (C), control rats exposed to hyperoxia (C + HOX), diabetic rats exposed to normoxia (T2D) and diabetic rats exposed to hyperoxia (T2D + HOX). To expose rats to hyperoxia, the hypoxia/hyperoxia chamber (SD‐EOS) was used, which was designed and built in our laboratory. This chamber has space for placing three rat cages (each cage: 35 × 25 × 14 cm in length, width, and height, respectively). The chamber is connected to oxygen and nitrogen tanks to regulate oxygen levels ranging from 1% to 99%. Rats in the C + HOX and T2D + HOX groups were exposed to hyperoxia (95% oxygen at an absolute pressure of 1 atm) for 2 h per day over 5 weeks (all weekdays except Fridays). Rats in the control and T2D groups were exposed to normoxia (21% oxygen at an absolute pressure of 1 atm) [11]. Our study's hyperoxia exposure duration was longer than previous reports, where the exposure typically ranges from 1 to 8 days (Table S1). In addition, a 35‐day lifespan in rats corresponds to about 2.5 years in humans and can be considered a long‐term intervention [31].

As shown in Figure 1, blood samples were collected from the tail of overnight fasted rats at week 0 (start of exposure to hyperoxia) and week 5 (end of exposure to hyperoxia). After blood clot formation, sera were centrifuged at 5000 ***g*** for 10 min and were used to measure glucose and insulin at week 0, and glucose, insulin, lactate and NO metabolites (NOx) at week 5. In addition, at week 5, SM (100 mg) was removed from anaesthetised rats (sodium pentobarbital, 60 mg/kg) and homogenised by homogeniser (Miccra D‐1, Germany) in a cold phosphate buffer (200 μL, 100 mM, pH 7.4) containing a protease inhibitor cocktail at a ratio of 1:5 (w:v) and then centrifuged for 10 min at 5000 g. The supernatant was used to measure NOx and lactate concentrations and protein levels of eNOS, iNOS and arginase. Animal body weights were measured (using the Tefal scale, sensitivity: 0.1 g) at week 0 and week 5.

### 2.3 Induction of Type 2 Diabetes

T2D was induced using the combination of a high‐fat diet (HFD) and low‐dose streptozotocin (STZ, 30 mg/kg, dissolved in citrate buffer, 0.1 mM, pH 4.5), as described previously [32]. Rats were fed an HFD, with a total caloric value of ~4900 kcal/kg, which consisted of 58.8% fat, 27% carbohydrate and 14.2% protein. After 3 weeks on HFD, rats were injected intraperitoneally with a single low dose of STZ [32]. The details of HFD preparation have been reported elsewhere [32]. Blood was collected from the tip of the tails of anaesthetised rats (sodium pentobarbital, 60 mg/kg), and fasting serum glucose (FSG) was measured one week post‐STZ injection; rats with FSG levels above 150 mg/dL were classified as type 2 diabetic rats. In the present study, sodium pentobarbital was used for anaesthetising animals, as it does not affect plasma glucose, insulin, free fatty acids and glucose tolerance, unlike ketamine/xylazine, isoflurane and chloral hydrate, which have been shown to do so [33, 34, 35]. In addition, unlike ketamine, which reduces NOS activity [36], sodium pentobarbital does not affect NOS activity [37].

### 2.4 Measurements

Serum glucose was measured using the glucose oxidase method (Pars Azmoon kit, Tehran, Iran). Serum insulin was measured using a rat‐specific insulin ELISA kit (Mercodia, Uppsala, Sweden). Intra‐ and inter‐assay coefficients of variation (CV) for glucose were 2.1% and 6.1%, respectively, and for insulin were 7.9% and 9.7%, respectively. Serum and tissue lactate levels were measured using the lactate dehydrogenase method with the Pars Azmoon kit (Tehran, Iran). Intra‐ and inter‐assay CVs for lactate were 1.9% and 2.2%, respectively. Protein concentration in the SM was assessed using the Bradford method [38]. In this study, total protein concentration in C, C + HOX, T2D and T2D + HOX groups were 1.9 ± 0.1, 1.5 ± 0.06, 2.1 ± 0.2 and 2.2 ± 1.2 mg/dL. The eNOS, iNOS and arginase protein levels in SM homogenates were measured in a single run using rat‐specific ELISA kits from ZellBio company. The sensitivity of the kits for eNOS, iNOS and arginase was 0.2, 0.2 and 0.078 ng/mL, respectively. eNOS, iNOS and arginase protein levels in SM are expressed as nmol/mg protein. All intra‐assay CVs were < 5%.

To measure the concentration of NOx, the Griess colorimetric method, described by Miranda et al. [39], was used with slight modification [40]. Briefly, zinc sulfate (15 mg/mL) was added to the samples for deproteinization, and the samples were centrifuged for 10 min at 10000 ***g*** at 4°C. Then, 100 μL of supernatant was added to microplate well, 100 μL vanadium (III) chloride (8 mg/mL in hydrochloric acid 1 M for reducing nitrate to nitrite), 50 μL sulfonamide (2% in 5% hydrochloric acid) and 50 μL N‐(1‐Naphthyl) ethylenediamine dihydrochloride (NEDD, 0.1% in distilled water) were added to each well. After 30 min incubation at 37°C, absorbance was read at 540 nm by an ELISA reader (Biotech, United States of America). The concentration of NOx in serum and SM was determined by a standard curve of nitrate (0–100 μM). Intra‐ and inter‐assay CV for NOx measurement were 2.1% and 2.2%, respectively.

### 2.5 Statistical Analysis

GraphPad Prism software (Version 8, La Jolla, California, USA) was used for data analyses. Data are presented as mean ± standard error of the mean (SEM). A one‐way analysis of variance (ANOVA) followed by the Tukey post hoc test was used to compare the serum and tissue NOx and lactate, as well as tissue protein levels of eNOS, iNOS and arginase between groups. A two‐way mixed analysis of variance followed by the Tukey post hoc test was used to compare the body weight, serum glucose and serum insulin between groups. In addition, the correlation between serum lactate and SM lactate was determined using Spearman correlation analysis. Two‐sided *p* < 0.05 was considered statistically significant.

## 3 Results

### 3.1 Effect of Hyperoxia on Serum Glucose, Insulin and NOx and Body Weight

As shown in Table 1, compared to controls, rats with T2D had significantly higher FSG at week 0 (81%, *p* < 0.001) and week 5 (135%, p < 0.001) as well as serum insulin (108%, *p* = 0.009) and body weight (11.2%, *p* < 0.05) at week 5. Hyperoxia did not affect FSG, serum insulin, and body weight in control rats. Compared to non‐treated T2D rats, T2D rats exposed to hyperoxia had lower FSG (28.0%, *p* < 0.001), insulin (36.2%, *p* = 0.023) and body weight (15.1%, *p* = 0.016) at week 5.

**TABLE 1:** Effect of hyperoxia on serum glucose, insulin, and body weight in the control and type 2 diabetic rats at week 0 and week 5.

	C		C + HOX		T2D		T2D + HOX	
	Week 0	Week 5	Week 0	Week 5	Week 0	Week 5	Week 0	Week 5
Serum glucose (mmol/L)	5.3 ± 0.1	4.6 ± 0.4	5.3 ± 0.1	4.4 ± 0.2	9.6 ± 0.2^###^	10.8 ± 0.5^###^	9.5 ± 0.1	7.8 ± 0.2***
Serum insulin (pmol/L)	116.1 ± 16.3	122.0 ± 27.6	120.7 ± 16.4	94.9 ± 7.4	135.0 ± 14.7	253.6 ± 32.5^###^	112.8 ± 14.5	161.9 ± 13.4*
Body weight (g)	253.8 ± 7.2	285.2 ± 10.2	249.8 ± 3.7	282.3 ± 5.8	265.7 ± 2.5	317.3 ± 12.4^#^	261.9 ± 5.1	269.3 ± 11.2***

*Note:* Values are mean ± SEM (*n* = 6). *p < 0.05, and ***p < 0.001 compared to the T2D at week 5. ^#^ *p* < 0.05 and ^###^ *p* < 0.001 compared to C group at their corresponding points.

Abbreviations: C + HOX, control rats exposed to hyperoxia; C, Control rats exposed to normoxia; T2D + HOX, diabetic rats exposed to hyperoxia; T2D, diabetic rats exposed to normoxia.

Serum NOx concentrations were comparable between control (34.5 ± 2.5 μM) and T2D (29.0 ± 2.4 μM) rats at week 5. Hyperoxia did not affect serum NOx in control rats (34.5 ± 2.5 vs. 31.8 ± 4.4 μM in C and C + HOX groups). Hyperoxia significantly (*p* = 0.005) decreased serum NOx concentration by 32.4% in the T2D + HOX group (19.6 ± 1.0 μM) compared to the T2D group (29.0 ± 2.4 μM).

### 3.2 Effect of Hyperoxia on Protein Levels of eNOS, iNOS and Arginase and NOx Concentration in the Soleus Muscle

**FIGURE 2:**
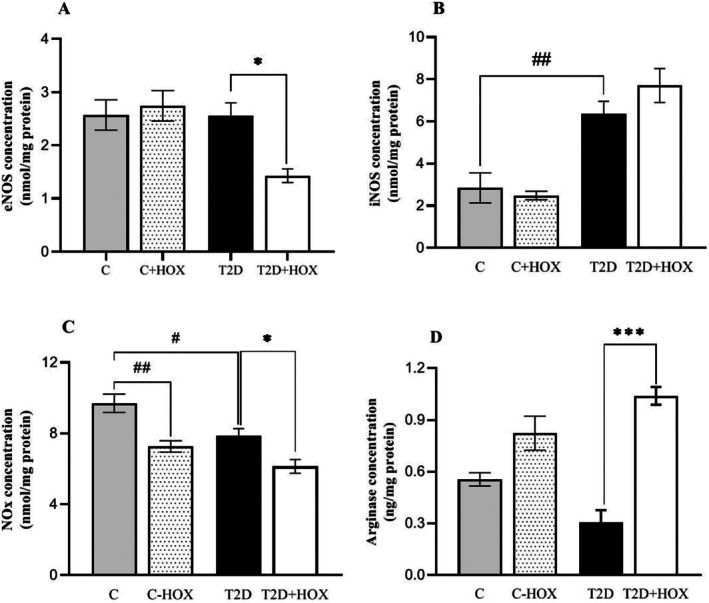
Effect of exposure to 95% oxygen (hyperoxia, HOX) on the protein levels of eNOS (A), iNOS (B), and NOx (C) and arginase (D) in soleus muscle of control and male rats with type 2 diabetes (T2D). eNOS, endothelial nitric oxide (NO) synthase; iNOS, inducible NO synthase; NOx, NO metabolites. C, Control rats exposed to normoxia; C + HOX, control rats exposed to hyperoxia; T2D, diabetic rats exposed to normoxia; T2D + HOX, diabetic rats exposed to hyperoxia. *p < 0.05, and ***p < 0.001 compared to the T2D. ^#^ *p* < 0.05 and ^##^ *p* < 0.01 compared to C group.

T2D rats had higher iNOS protein (123%, *p* = 0.003) and lower NOx levels (19.7%, *p* = 0.029) but comparable eNOS and arginase proteins to controls in the SM (Figure 2). Exposure to hyperoxia did not affect eNOS, iNOS and arginase levels but decreased NOx (25%, *p* = 0.003) in control rats (Figure 2). In T2D rats, exposure to hyperoxia decreased eNOS protein levels (46.2%, *p* = 0.002, Figure 2A) in SM but did not affect iNOS levels (Figure 1B). In addition, it decreased NOx levels (22.8%, *p* = 0.011, Figure 2C) and increased arginase (2.3‐fold, *p* < 0.001, Figure 2D).

### 3.3 Effect of Hyperoxia on Lactate Level in Serum and Soleus Muscle

**FIGURE 3:**
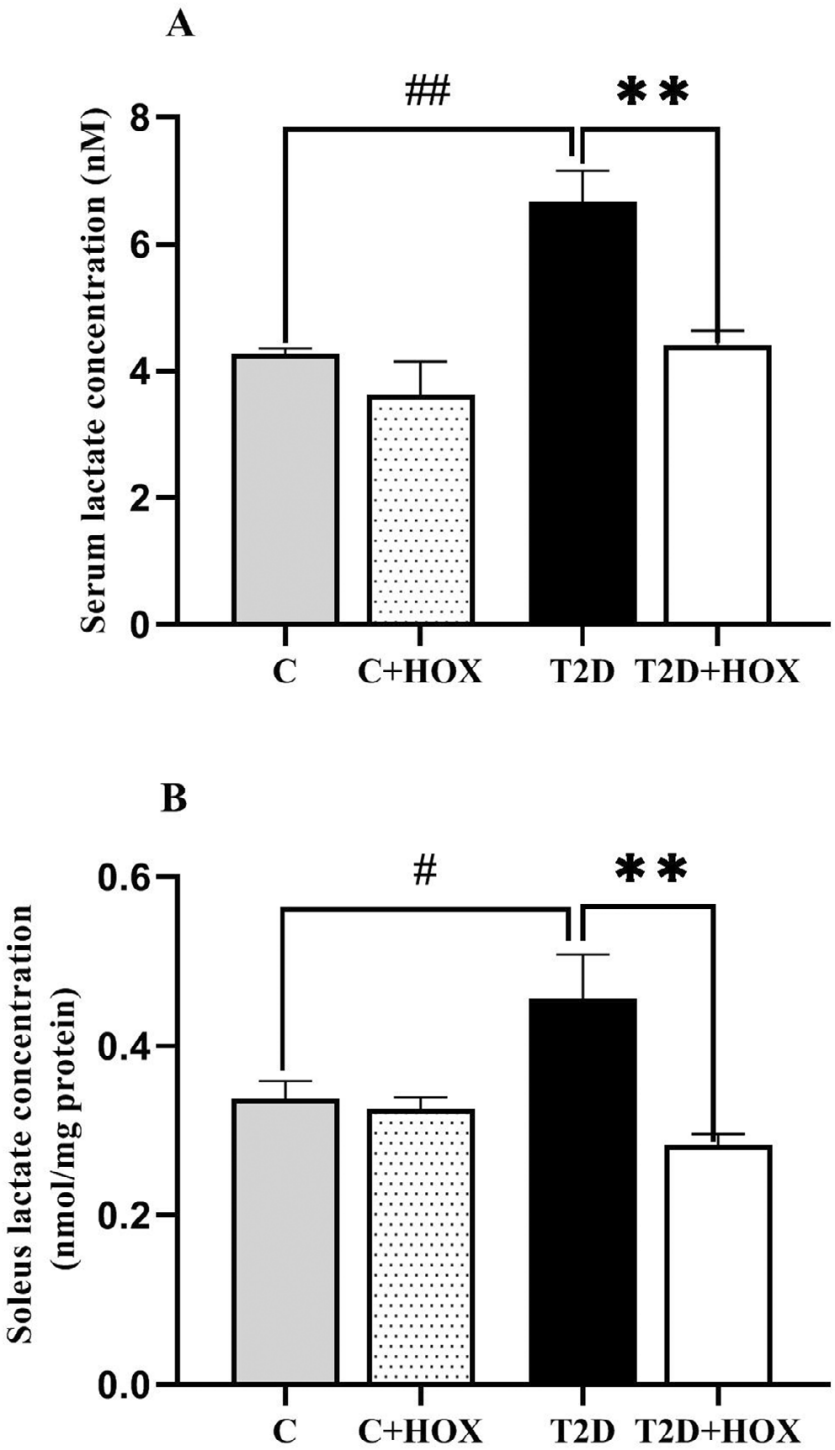
The effect of exposure to 95% oxygen (hyperoxia, HOX) on the lactate level in the serum (A) and soleus muscle (B) of control and male rats with type 2 diabetes (T2D). C, Control rats exposed to normoxia; C + HOX, control rats exposed to hyperoxia; T2D, diabetic rats exposed to normoxia; T2D + HOX, diabetic rats exposed to hyperoxia. **p < 0.01 compared to the T2D; ^#^ *p* < 0.05, and ^##^ *p* < 0.01 compared to the C group. Values are mean ± SEM (*n* = 6).

**FIGURE 4:**
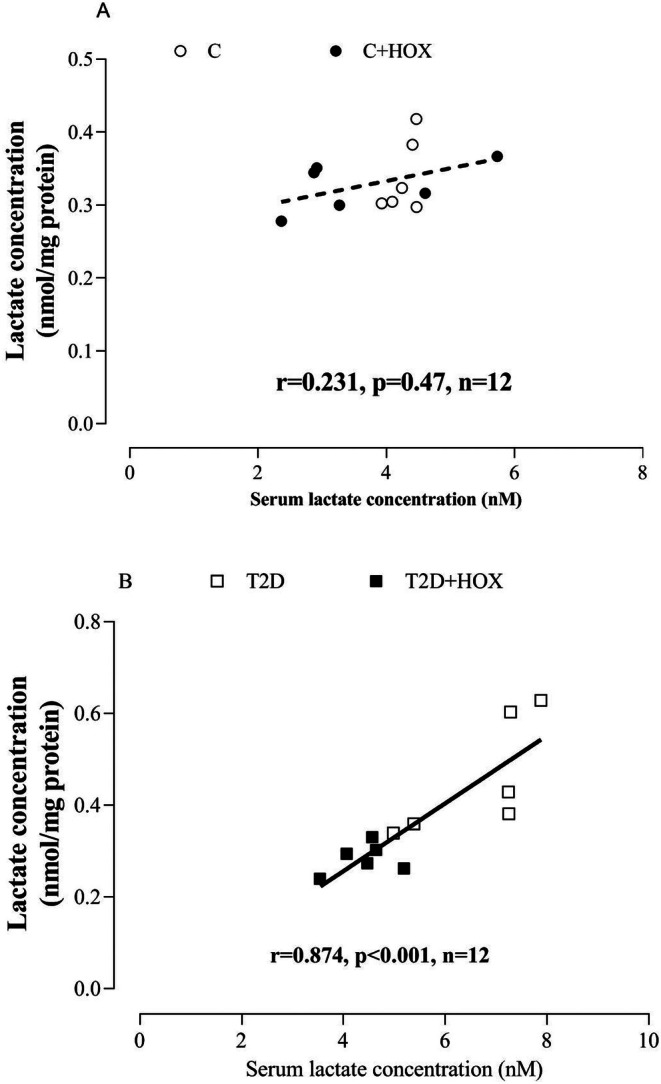
Correlation of serum lactate with soleus lactate concentration in control (A) and rats with type 2 diabetes (T2D) (B). C, Control rats exposed to normoxia; C + HOX, control rats exposed to hyperoxia; T2D, diabetic rats exposed to normoxia; T2D + HOX, diabetic rats exposed to hyperoxia; n, number.

At the end of the study, serum lactate (Figure 3A) and SM lactate (Figure 3B) in T2D rats were higher than in controls by 56% (*p* = 0.001) and 35% (*p* = 0.046). Hyperoxia did not affect serum and tissue lactate in control rats. In T2D rats, hyperoxia decreased serum lactate and SM lactate by 34% (*p* = 0.002) and 38% (*p* = 0.009). In addition, serum lactate was positively correlated with SM lactate (*r* = 0.847, *p* < 0.001) in diabetic rats but not in control rats (Figure 4).

## 4 Discussion

This study showed that exposure to hyperoxia (95% oxygen for 5 weeks) decreased NO bioavailability in the SM of male rats with T2D, as indicated by decreased eNOS (~46%) and NOx concentrations (~23%) and increased arginase (230%). These findings indicate that decreased NO bioavailability is a side effect of oxygen therapy in rats with T2D.

In line with our previous reports, hyperoxia decreased fasting glucose and insulin levels as well as body weight in T2D rats [11, 41]. These beneficial effects of hyperoxia on carbohydrate metabolism are due to reducing abdominal fat and increasing the browning of white adipose tissue [11, 41]. In our study, iNOS protein was higher, and NOx levels were lower in the SM of T2D than in the control group. However, eNOS and arginase levels in SM, as well as serum NOx levels, were similar to values observed in controls. In line with these findings, increased iNOS protein and decreased NOx levels in the SM have been reported in T2D rats after 2 [42] and 6 months [15]. Decreased NOx levels in the soleus muscle, despite increased iNOS, seem to be paradoxical at first glance. However, in addition to changes in NOS isoforms that govern NO production, the amount of NOx is influenced by NO metabolism, including reverse relation with oxygen concentration and also quenching of NO by increased oxidative stress, which is observed in diabetic rats [41]. Despite increased iNOS expression, decreased NOx levels in diabetic rats in our study may be due to increased NO metabolism, suggesting that increased iNOS expression may be a compensatory response to decreased NO bioavailability, which has been reported in different tissues of animals with diabetes [43, 44], including soleus skeletal muscle [15, 40]. This notion is supported by a recent study that indicates, despite the traditional view that considers merely a pathological role for iNOS, it may have a protective intermediatory role in situations such as diabetes [45]. In addition, skeletal muscle may act as a nitrate reservoir that exports nitrate to other tissues during NO deficiency states, such as diabetes [46]. Evidence that supports this notion is decreased nitrate levels as well as increased mRNA expression of sialin, a nitrate transporter, in the soleus muscle of rats with T2D [40]. Interestingly, nitrate administration restored elevated sialin expression in the soleus muscle of rats with T2D [47], indicating the participation of nitrate stores in the skeletal muscle to meet the needs of other body tissues.

The literature regarding serum and tissue NOx [15, 42, 48, 49, 50, 51] and tissue eNOS [15, 42, 51] in T2D is inconsistent and appears to depend on the duration of T2D, where no change in serum NOx has been reported 1 [50], 2 [42, 50] and 3 [51] months after induction of T2D, but decreased serum and tissue NOx and tissue eNOS have been reported after 6 months [15].

In this study, rats with T2D had higher serum (56%) and SM (35%) lactate levels than controls, and a positive correlation between serum and SM lactate was observed, indicating that in T2D, as a hypoxic state, anaerobic metabolism increases. Elevated serum lactate levels in individuals with T2D [52] contribute to insulin resistance and the progression of T2D [53]. In our study, hyperoxia decreased serum and lactate levels in SM by 34% and 38%, indicating a decrease in anaerobic metabolism following oxygen therapy. In this context, exercise combined with inhalation of 60% oxygen for 40 min in human males aged 19–25 years resulted in a 27.8% decrease in lactate levels in skeletal muscles [54]. In addition, exposure to 95% oxygen for 24 h leads to a 50% reduction in lactate release from 3 T3‐L1 adipocytes [55]. In line with our results, exposure to 95% oxygen decreased eAT lactate concentrations by 50% [20] and serum lactate by 22% [11] in male rats with T2D. Therefore, hyperoxia may improve insulin sensitivity by reducing serum and tissue lactate levels.

In this study, exposure to 95% oxygen resulted in a ~46% decrease in eNOS protein levels and a ~23% decrease in NOx levels but did not affect iNOS levels in SM of rats with T2D. The effects of hyperoxia on eNOS, iNOS and NOx levels vary across the examined tissues (See Table S1). In line with our results, most studies have reported decreased eNOS expression following exposure to hyperoxia, as indicated in in vitro [56] and in vivo studies [20, 21, 22, 57]. However, some studies have reported no change [58] or increased eNOS expression following exposure to hyperoxia in the lung [59, 60, 61]. Our results that exposure to hyperoxia decreases soleus muscle NOx levels are in line with the previous reports in astrocytes [22], liver [18], eye [22], eAT [20] and lung [19]. However, some studies reported increased tissue NOx in the lung following exposure to hyperoxia [21, 25]. The effect of hyperoxia on iNOS expression is controversial, with no change [18, 19, 20, 21, 22], decreases [22, 23] and increases [24, 61] having been reported across different tissues. The duration of hyperoxia, the animal age, and the specific tissues studied partially explain the discrepancies observed in eNOS and iNOS levels following exposure to hyperoxia. One‐day exposure to hyperoxia decreased iNOS levels in the heart tissue of pigs with hemorrhagic shock [23], whereas long‐term exposure (2.5–35 days) resulted in either an increase [24] or no change in iNOS level [18, 19, 20, 21]. In addition, long‐term exposure to hyperoxia (5 [21], 7 [22] and 35 [20] days) reduced eNOS expression, while short‐term exposure (3.5 days [18]) had no significant effect. In addition, hyperoxia did not alter eNOS levels in the liver [18] but decreased it in the lung [21], eye [22] and eAT [20]. Animal age is another factor that may explain variability in the effects of hyperoxia on NOS expression [62]. Exposure to hyperoxia in juvenile rats (age 21–29 days) [60] increased eNOS, but in mice aged 42–56 days [21] and adolescent rats (56 [22] and 60 [20] days) as well as in an in vitro study [56] decreased eNOS expression. Another issue that may explain different responses of NOS isoforms to hyperoxia is different K_m_ values for O_2_, which are 23 μM (~17 mmHg) for eNOS, 135 μM (~100 mmHg) for iNOS and 350 μM (~265 mmHg) for nNOS [17]. Normal tissue O_2_ levels range from 15 to 100 mmHg [63], indicating an order of sensitivity to O_2_ availability as nNOS > iNOS > eNOS [64]. In the current study, hyperoxia did not affect the parameters related to NO metabolism in control rats, except for decreased NOx levels in SM. The likely explanation is an inverse correlation between NO half‐life and O_2_ concentration [16, 17], representing rapid NO metabolism under hyperoxia [17].

This study found that exposure to 95% oxygen for 35 days increased arginase protein levels in SM by 2.3‐fold. Consistent findings in the literature (Table S1) show that hyperoxia increases arginase levels in the eAT of diabetic rats [20], lung of healthy mice [19] and liver of healthy rats [18]. When mice were exposed to 95% oxygen for 96 h, liver arginase expression tripled, with a 50% increase in liver ornithine but no change in citrulline [18]. In addition, exposure to 100% oxygen for 60 h led to a 64% increase in arginase activity in rat lungs [19].

Hyperoxia produces reactive oxygen species (ROS), resulting in oxidative stress in diabetic rats [41]. Oxidative stress decreases eNOS expression and activity [65] by decreasing tetrahydrobiopterin (BH4), an essential cofactor for eNOS [5], and upregulates arginase [66, 67, 68] by PKC‐mediated activation of the RhoA/Rho kinase pathway [69]. Increased arginase expression or activity competes with eNOS for the substrate *L*‐arginine [70] and causes eNOS uncoupling [68], which exacerbates oxidative stress, further decreasing NO bioavailability. Notably, arginase deficiency prevents oxidative stress and NOS‐dependent ROS formation and preserves NO availability in mice with oxygen‐induced retinopathy [66]. Hyperoxia increases arginase protein expression, which converts *L*‐arginine into urea and *L*‐ornithine. Since arginase is ~1000 times more effective than NOS at using *L*‐arginine, increased arginase activity limits *L*‐arginine availability for NOS [71, 72, 73]. This may account for decreased NO levels in skeletal muscle, similar to what was previously reported for the liver [18] and eAT [20]. In addition, reduced NO bioavailability following hyperoxia might result from higher NO clearance and decreased *L*‐arginine metabolism via hepatic NOS pathways due to lower blood flow in the liver [18].

Reduced NO bioavailability is a key mechanism underlying vascular insulin resistance in T2D and is an independent predictor of new‐onset T2D [14]. It also is a hallmark of endothelial dysfunction, an early step in the development of atherosclerosis, hypertension and cardiovascular diseases [74]. It is, therefore, necessary to take into account decreased NO bioavailability when hyperoxia is used in patients with T2D.

Our study has some strengths. First, our animal model of T2D (HFD to induce obesity and insulin resistance combined with a low dose of STZ to cause the partial destruction of pancreatic beta cells [75]) displays stable hyperglycemia and insulin resistance [11] and exhibits characteristics similar to those of obese T2D in humans [75]. It should be mentioned that 65%–85% of patients with T2D are overweight or obese [76, 77, 78]. Second, the duration of hyperoxia exposure in our study (35 days) is longer than in other studies (Table S1), which mostly range from 1 to 7 days. Our study has some limitations. First, our study was limited to male rats to avoid the influence of hormonal variations during the estrous cycle. The preference for using male animals over females is common in the field of endocrinology, where 66% of animal research involves males [32, 79]. However, it is unlike the NIH recommendation that advocates for balancing both sexes in preclinical studies [80]. Second, like other previous reports, we did not measure nNOS protein (see Table S1). Finally, from the results of this study, we cannot determine NOS isoforms involved in NOx production, as we did not conduct pharmacological studies.

## 5 Conclusion

Hyperoxia decreased NO bioavailability in the SM of male rats with T2D. This reduction was associated with decreased eNOS and NOx and increased arginase levels in the SM. These findings indicate that the previously reported antihyperglycemic effect of hyperoxia in rats with T2D [11, 41] is accompanied by NO deficiency in the SM as an adverse effect of hyperoxia.

## Author Contributions

M.M., S.J., R.N. and A.G. designed the experiments; M.M., S.J. and R.N. performed experiments and collected data; S.J. and A.G. discussed the results and strategy; A.G. supervised, directed and managed the study; M.M., S.J. and A.G. drafted the article. M.M., S.J., R.N. and A.G. final approved the version to be published.

## Conflicts of Interest

The authors declare no conflicts of interest.

## Supporting information


**Table S1:** A summary of in vitro and in vivo animal studies assessed the effect of hyperoxia on nitric oxide metabolism in different tissues.

## Data Availability

Some or all datasets generated during and/or analysed during the current study are not publicly available but are available from the corresponding author on reasonable request.
