# Supplementary material for: The Effect of Hyperoxia on Nitric Oxide Metabolism in the Skeletal Muscle of Male Type 2 Diabetic Rats
Source: Endocrinol Diabetes Metab. 2025 Aug 4;8(5):e70090. doi: 10.1002/edm2.70090 (PMC12319423; doi:10.1002/edm2.70090)
Supplement: Supplementary file 1 — Table S1: A summary of in vitro and in vivo animal studies assessed the effect of hyperoxia on nitric oxide metabolism in different tissues. [file EDM2-8-e70090-s001.docx]

| **Supplementary Table 1.** **A summary of *in vitro* and *in vivo* animal studies assessed the effect of hyperoxia** **on nitric oxide metabolism in different tissues** | | | | | | | | | | | | |
| --- | --- | --- | --- | --- | --- | --- | --- | --- | --- | --- | --- | --- |
| **Study** | **Year** | **Species** | **Model** | **Sex** | **Age (days)** | **Duration (days)** | **Exposed oxygen (%)** | **Cell/tissue** | **eNOS** | **iNOS** | **NO_X_** | **Arginase** |
| ***In vitro*** |  |  |  |  |  |  |  |  |  |  |  |  |
| Zhang et al. [^56^](#_ENREF_56) | 2019 | – | - | – | – | NR | 85 | PMVECs  cell line | ↓ | NR | NR | NR |
| Dong et al. [^22^](#_ENREF_22) | 2022 | – | - | – | – | 1 | 75 | Primary cultured astrocytes | NR | ↓ | ↓ | NR |
|  |  |  |  |  |  | 3 |  |  |  |  |  |  |
|  |  |  |  |  |  | 7 |  |  |  | ↔ |  |  |
| North et al. [^59^](#_ENREF_59) | 1996 | - | - | NR | - | 2 | 20 | Primary cultured PAEC | ↑ | NR | NR | NR |
| ***In vivo*** |  |  |  |  |  |  |  |  |  |  |  |  |
| Huang et al [^57^](#_ENREF_57) | 2021 | Mice | H | NR | 1 | 7 | 85 | Lung | ↓ | NR | NR | NR |
| Edgar et al. [^58^](#_ENREF_58) | 2015 | Mice | H | NR | 7 | 6 | 75 | Retinal | ↔ | NR | NR | NR |
| Arkovitz et al. [^21^](#_ENREF_21) | 1997 | Mice | H | F | 42-56 | 5 | 95 | Lung | ↓ | ↔ | ↑ | NR |
| Jin et al. [^61^](#_ENREF_61) | 2019 | Mice | H | F/M | 56-84 | 2.5 | >95 | Lung | ↑ | ↑ | NR | NR |
| Bhandari et al. [^24^](#_ENREF_24) | 2011 | Mice | H | NR | 28-32 | 2.5 | 100 | Lung | NR | ↑ | NR | NR |
| Malleske et al. [^18^](#_ENREF_18) | 2006 | Mice | H | NR | 42 | 3.5 | >95 | Liver | ↔ | ↔ | ↓ | ↑ |
| Bailey et al. [^25^](#_ENREF_25) | 2002 | Mice | H | NR | NR | 2 | >90 | Lung | NR | NR | ↑ | NR |
| Potter et al. [^60^](#_ENREF_60) | 1999 | Rats | H | NR | 21-29 | 8 | >95 | Lung | ↑ | NR | NR | NR |
| Dong et al. [^22^](#_ENREF_22) | 2022 | Rats | H | M | 56 | 7 | 75 | Eye | ↓ | NR | ↓ | NR |
| Ghasemi et al. [^20^](#_ENREF_20) | 2023 | Rats | T2D | M | 60 | 35 | 95 | eAT | ↓ | ↔ | ↓ | ↑ |
| Que et al. [^19^](#_ENREF_19) | 1998 | Rats | H | M | Adults | 2.5 | 100 | Lung | NR | ↔ | ↓ | ↑ |
| Datzmann et al. [^23^](#_ENREF_23) | 2019 | Pigs | HS | F/M | 500-600 | 1 | 100 | Heart | NR | ↓ | NR | NR |
| The evidence presented are based on Scopus and PubMed using "hyperoxia" and "nitric oxide synthase" as keywords. In addition, reference lists of the retrieved articles were examined to identify further relevant studies. eAT, epididymal adipose tissue; eNOS, endothelial nitric oxide (NO) synthase; F, female; H, healthy; HS, hemorrhagic shock; iNOS, inducible NOS; M, male; NOx, NO metabolites; NR, not reported; PAEC, ovine fetal intrapulmonary artery endothelial cells; PMVECs, pulmonary microvascular endothelial cells; T2D, type 2 diabetes. ↓, decrease; ↑, increase; ↔, no change. | | | | | | | | | | | | |
